# Supplementary material for: Noise robustness of persistent homology on greyscale images, across filtrations and signatures
Source: PLoS One. 2021 Sep 24;16(9):e0257215. doi: 10.1371/journal.pone.0257215 (PMC8462731; doi:10.1371/journal.pone.0257215)
Supplement: S1 Appendix — (PDF) [file pone.0257215.s001.pdf]

S1 Appendix

Table 1 summarizes the notation. For the purpose of clarity, throughout the manuscript we denote spaces with capitals, vectors with bolded lower case, scalars with lower case (with the exception of the standard notation C for the SVM regularization paper, referred briefly in the paper), and functions with the Greek alphabet lower cases (with the exceptions of the standard notation for Wasserstein W_p, and l_p and L_p distances).

Table 1. Important notation and acronyms.

Notation	Interpretation
S	space
$S_{r_1} \subseteq S_{r_2} \subseteq \cdots \subseteq S_{r_t}$	filtration (S_r approximates S at resolution, scale or time $r \in \mathbb{R}$)
$Z = [z_{uv}]$	image as a two-dimensional matrix, z_{uv} is the greyscale value of pixel (u, v)
z_0	threshold greyscale value (to obtain binary image)
n_x	number of pixels in x direction
n_y	number of pixels in y direction
$X \subset \mathbb{R}^n$	point cloud
$VR(X, r)$	Vietoris-Rips simplicial complex with resolution r
$\delta_X : \mathbb{R}^n \to \mathbb{R}$	distance (filtration) function
$\delta_{X,m} : \mathbb{R}^n \to \mathbb{R}$	distance-to-a-measure (DTM) (filtration) function with parameter m
K	cubical complex
$\phi_{z_0} : K \to \mathbb{R}$	binary filtration function with parameter z_0
$\phi_{\text{grsc}} : K \to \mathbb{R}$	greyscale filtration function
$\phi_{d_0,z_0} : K \to \mathbb{R}$	density filtration function with parameters d_0 and z_0
$\phi_{(u_0,v_0),z_0} : K \to \mathbb{R}$	radial filtration function with parameters (u_0, v_0) and z_0
PH	persistent homology, information about k-dimensional cycles
(b_i, d_i)	persistence interval, i.e., pair of birth and death values for cycle i
$l_i = d_i - b_i$	lifespan or persistence of a cycle i
PD	persistence diagram
W_p	Wasserstein distance between PDs (referred to as bottleneck distance for $p = +\infty$)
λ	persistence landscape (as a function)
PL	vectorized persistence landscape
PI	persistence image
ρ	PI weight function
