# Supplementary material for: Noise robustness of persistent homology on greyscale images, across filtrations and signatures
Source: PLoS One. 2021 Sep 24;16(9):e0257215. doi: 10.1371/journal.pone.0257215 (PMC8462731; doi:10.1371/journal.pone.0257215)
Supplement: Attachment — Submitted filename: review_turkes+_manuscript.pdf [file pone.0257215.s002.pdf]

Review

PLOS ONE - Turkes et al.

June 24, 2021

Title. Noise robustness of persistent homology on greyscale images, across filtrations and signatures.

Authors. Renata Turkě, Jannes Nys, Tim Verdonck, Steven Latré,

Purposes. Gaining insights on the stability of persistent homology in practice (on the MNIST dataset).
Authors address image classification under typical forms of noise.

Results. The stability is influenced by the considered filtration and signature (input and output).
In classification, PH features are not in general stable to noise.

Typos.

- **line 115:** analog of → analog to (moreover in british, analogue is better, analog is more convenient for electronic circuits)
- **line 244, 270, 314, etc...** weighing → weight (more standard)
- **Caption of Figure 3:** 0-dimensional → 1-dimensional

Definitions and notations.

- **line 20:** why "scalars"? sets, functions, vectors already capture PD, PL, and PI. By "scalars", I don't understand which PH signature the authors refer to.
- **line 101:** better to avoid the notation for round up numbers that was never introduced. Plain words are better.
- **line 106:** $[z_{uv}]$ is not standard for matrices though consistent all along the paper.
- **lines 108–110:** $X(Z)$ depends on z_0 which is not encoded in the symbol.

- **line 126 and between lines 135-136:** ϕ is both the general filtration function and the specific grayscale filtration function. I suggest that the general function has no symbol assigned or is named otherwise
- **line 161:** symbol d already used for threshold distance. Suggestion: change d at between lines 140-141 into something else
- **Caption of Table 2:** title of second column not clear
- **Table 3:** column names should be highlighted, for example in bold
- **Figure 2 and 3 at pages 14-15:** better to display differently since they interrupt items giving problems in reading.
- **Table 5, Table 4, Figure 2 and 3 at pages 14-15:** salt and pepper parameters 5 and 10, as well as other labels, are not clear to unaccustomed readers. It is difficult to specify it in the caption. I suggest to specify it in the items of Section Noise Robustness at page 13.
- **Caption of Table 4, Table 5:** Specify the connection with the table rows (in particular the first) as done in Figure 2 and Figure 3
- **Table 5:** the color channel should be linked to a scale range otherwise the discussion at page 18 is difficult to follow (in particular 35%, 10%, etc...)
- **line 465:** symbol W_2 for Wasserstein distance but it was previously introduced as ω_2
- **line 490:** C already used for cubical complexes. Not necessary to change. Better to disambiguate when the parameter C is introduced.

Bibliography.

- **Ref. 50:** add a blank space in front of "Available at". The url is necessary?
- **Multiple Refs:** to be fixed the endings with ";." due to missing fields in the bibtex.
