# Supplementary material for: Noise robustness of persistent homology on greyscale images, across filtrations and signatures
Source: PLoS One. 2021 Sep 24;16(9):e0257215. doi: 10.1371/journal.pone.0257215 (PMC8462731; doi:10.1371/journal.pone.0257215)
Supplement: Attachment — Submitted filename: response_to_reviewers_turkevs2021noise.pdf [file pone.0257215.s003.pdf]

Dear editor and reviewers,

Thank you for your valuable comments for our manuscript PONE-D-21-14381 entitled “Noise robustness of persistent homology on greyscale images, across filtrations and signatures”.

In the remainder of this letter, we address each of your comments separately. We also attach the improved version of the paper, with all changes compared to the original manuscript highlighted (any removed words are crossed out with a single line and coloured red, whereas any added words are underlined with a squiggle and coloured blue).

We also made some minor changes related to the typesetting and formatting (e.g., since the submission guidelines state that tables cannot include images, some of our tables have been changed to figures, and the figures are now separately uploaded as .tiff files), so that the manuscript now completely satisfies the PLOS One journal requirements.

Thank you once again for the time and effort you invested going through our manuscript in detail, and for all the suggestions that helped to further improve our work.

Sincerely,
Renata Turkeš

Antwerp, Belgium, August 10th, 2021

Response to the comments from Reviewer #1

Reviewer #1, General comment

Purposes. Gaining insights on the stability of persistent homology in practice (on the MNIST dataset). Authors address image classification under typical forms of noise.

Results. The stability is influenced by the considered filtration and signature (input and output). In classification, PH features are not in general stable to noise.

The paper is interesting and deals with relevant issues in TDA. Our suggestion and observations are included in the attached pdf. There is a list of minor improvements after which I think the paper will be suitable for publication on PLOS.

Thank you for encouraging words. We respond to each of your suggestions below.

Reviewer #1, Comment #1

Typos:

- line 115: analog of → analog to (moreover in British, analogue is better, analog is more convenient for electronic circuits)
- line 244, 270, 314, etc... weighing → weight (more standard)
- Caption of Figure 3: 0-dimensional → 1-dimensional

All typos are corrected, thank you so much once again for reading the manuscript in such detail!

Reviewer #1, Comment #2 Definitions and notations, Remark #1

line 20: why "scalars"? Sets, functions, vectors already capture PD, PL and PI. By "scalars", I don't understand which PH signature the authors refer to.

You are definitely right that sets, functions and vectors already capture PD, PL and PI (which we use, as explained in the manuscript, as they are the most common signatures and sufficient to tell our story). With this sentence, we only want to highlight that there are many very different ways to represent persistent homology information.

In the end of the section, we had listed a number of other examples of signatures, including scalars, but we now make this more clear:

> Some other persistence signatures have been introduced in the literature, such as: Betti numbers (across scales) [10, 15]; silhouette [6], which combines all layers of persistence landscape functions into a single, weighted average function, with greater weight assigned to features with longer lifespans [3]; persistence intensity function [7], which is evaluated on a grid to obtain a ~~PI [3]; algebraic function of b_i and $d_i - b_i$, e.g., $\sum b_i^p(d_i - b_i)^q$, so that p and q determine the importance of some of the qualities about cycles (e.g., size of holes) [1];~~ persistance image [3]; or Euler characteristic, the difference between the number of connected components and the number of holes (across scales) [12]. As already indicated in the introduction, persistent homology information can also be summarized with a scalar, for instance with: amplitude, distance from empty persistence diagram [9]; entropy, a real number calculated using the lifespans of all features [9], which thus only depends on the persistence but not on the particular birth or death times; ~~Betti numbers (across scales) [10, 15]; or Euler characteristic, the difference between the number of connected components and the number of holes(across scales) [12]~~or an algebraic function of b_i and $d_i - b_i$, e.g., $\sum b_i^p(d_i - b_i)^q$, so that p and q determine the importance of some of the qualities about cycles (e.g., size of holes) [1]
> .

Reviewer #1, Comment #2 Definitions and notations, Remark #2

line 101: better to avoid the notation for round up numbers that was never introduced. Plain words are better.

We agree that the ceiling notation made the paragraph hard to read, and have therefore rephrased it in the following way:

> To circumvent this issue, [5] propose to rather consider distance-to-a-measure (DTM) $\delta_{X,m}$ as the filtration function, which is defined as the average ~~of distances from $\lceil m|X|\rceil$~~ distance from a given number of nearest neighbours in X (and is thus a smooth version of the distance function) [2]. The number of neighbours that are considered is determined by the parameter m, which represents the percentage of the total number of point cloud X points. In our computational experiments, we will quantify how much robustness to noise is actually gained in practice with PH on the DTM (with ~~$m = 0.1$~~ $m = 0.1$, a commonly suggested and typically default value) compared to the Rips filtration.

Reviewer #1, Comment #2 Definitions and notations, Remark #3

line 106: $[z_{uv}]$ is not standard for matrices though consistent all along the paper.

This is the notation that we are most used to and that we found the simplest, that is also used e.g., in Horn, Roger A., and Charles R. Johnson. *Matrix analysis*, Cambridge University Press, 2012, page 5, (with 37 956 citations!), or on Wikipedia (although with parentheses, that we refrained from as they are used throughout the paper for different purposes).

Reviewer #1, Comment #2 Definitions and notations, Remark #4

lines 108-110: $X(Z)$ depends on z_0 which is not encoded in the symbol.

Thank you for pointing this out, we changed the notation to $X(Z, z_0)$ throughout the paper.

Reviewer #1, Comment #2 Definitions and notations, Remark #5

line 126 and between lines 135-136: ϕ is both the general filtration function and the specific grayscale filtration function. I suggest that the general function has no symbol assigned or is named otherwise.

We use the general function ϕ also in lines 112-125 to describe the notions of filtration function on a cubical complex and the resulting filtration, and therefore keep this symbol. As can be seen more clearly from the Notation table in Appendix A, the filtration functions on cubical complexes are all represented using ϕ, but now in a unique way: ϕ_{z_0}, ϕ_{grsc}, ϕ_{d_0,z_0} and $\phi_{(u_0,v_0),z_0}$ represent respectively the binary, greyscale, density and radial filtration function.

Reviewer #1, Comment #2 Definitions and notations, Remark #6

line 161: symbol d already used for threshold distance. Suggestion: change d at between lines 140-141 into something else.

Indeed, d was used to denote both the distance threshold for the density filtration function, and the death value in a persistence interval, thank you for bringing this to our attention. Since it is standard in the literature to use d to denote both distances and persistence death values, we keep d as the symbol for death values, but use d_0 as the threshold distance. We note that this can never lead to any confusion, since we now also explicitly mention:

> The cardinality of this multi-set of persistence intervals (b_i, d_i) $(i \in \mathbb{N}^+)$ counts the number of k-dimensional cycles (...)

Reviewer #1, Comment #2 Definitions and notations, Remark #7

Caption of Table 2: title of second column not clear

The title of the second column has been changed, and we now also include a more detailed description in the table caption (instead of d, we now also use the Greek alphabet lower case δ for general distance function):

Table 1. **Persistent homology across signatures.**

**Persistence signature**	**Limiting behaviour of** $\delta^2(PH, \emptyset)$
PD	$O(l^2)$
PL	$O(l^3)$
PI, with weight function $\rho(b, l) = l^2$	$O(l^4)$

The choice of persistence signature, and the corresponding metric, determines how sensitive PH is to cycles (b, d) with short persistence, or lifespan, $l = d - b$. This influences the noise robustness and discriminative power of PH. The table lists the importance of a cycle with lifespan l, for the Wasserstein w_2 limiting behaviour, or growth rate, of the function $\delta^2(PH, \emptyset) = \delta^2(\{(b, d)\}, \emptyset) = f(d - b) = f(l)$, where distance δ represents the Wasserstein W_2 distance between persistence diagrams, or l_2 distance between persistence landscapes or persistence images. The growth rate reflects the importance of a cycle with lifespan l, which influences the noise robustness and discriminative power of PH.

Reviewer #1, Comment #2 Definitions and notations, Remark #8

Table 3: column names should be highlighted, for example in bold

Done! We also made these changes in all the other tables (and according to the PLOS One template and guidelines), so thanks for noticing this!

Reviewer #1, Comment #2 Definitions and notations, Remark #9

Figure 2 and 3 at pages 14-15: better to display differently since they interrupt items giving problems in reading.

These figures (now uploaded separately as .tiff files, according to the PLOS One guidelines) are now only placed after the long paragraph with items that discusses them in detail.

Reviewer #1, Comment #2 Definitions and notations, Remark #10

Table 5, Table 4, Figure 2 and 3 at pages 14-15: salt and pepper parameters 5 and 10, as well as other labels, are not clear to unaccustomed readers. It is difficult to specify it in the caption. I suggest to specify it in the items of Section Noise Robustness at page 13.

Done!

Reviewer #1, Comment #2 Definitions and notations, Remark #11

Caption of Table 4, Table 5: Specify the connection with the table rows (in particular the first) as done in Figure 2 and Figure 3.

Done!

Reviewer #1, Comment #2 Definitions and notations, Remark #12

Table 5: the color channel should be linked to a scale range otherwise the discussion at page 18 is difficult to follow (in particular 35%, 10%, etc...)

Done!

Reviewer #1, Comment #2 Definitions and notations, Remark #13

line 465: symbol W_2 for Wasserstein distance but it was previously introduced as ω_2

Thank you for noticing this discrepancy. As noted in the appendix, we use Greek alphabet lower cases as symbols for functions. However, since we made the exception for the L_p and l_p distances, we now also do the same for Wasserstein distance and use the standard literature notation W_p throughout the paper. We now also clarify this in the appendix:

> Table 4 summarizes the notation. For the purpose of clarity, throughout the manuscript we denote spaces with capitals, vectors with bolded lower case, scalars with lower case ~~of the Latin alphabet ($x, y \in \mathbb{R}$, $\mathbf{x}, \mathbf{y} \in \mathbb{R}$~~(with the exception of the standard notation C for the SVM regularization paper, referred briefly in the paper), and functions with the Greek alphabet lower cases (with the exceptions of the standard notation for Wasserstein W_p, and l_p and L_p distances).

Reviewer #1, Comment #2 Definitions and notations, Remark #14

line 490: C already used for cubical complexes. Not necessary to change. Better to disambiguate when the parameter C is introduced.

Throughout the revised manuscript, we now use K to denote cubical complexes. We think that the choice of K will make it less likely for the reader to assume a scalar, or a constant, for which it is standard to use notation C or c (that we keep, respectively for the SVM regularization parameter and the constant in the stability theorems).

Reviewer #1, Comment #3

Bibliography:

- Ref. 50: add a blank space in front of "Available at". The url is necessary?
- Multiple Refs: to be fixed the endings with ";." due to missing fields in the bibtex.

Done!

Response to the comments from Reviewer #2

Reviewer #2, General comment

The paper presents how noise in data affects its persistent homology. It clearly explains the relevance of the problem and how the results compare with existing literature.

The authors discuss how different choices of filtrations and noise on the MNIST handwritten digits dataset perturb the persistent signatures (persistence diagram, persistent images and persistent landscapes). This presents a useful practical example of how the theoretical stability theorem might or not work in practice. The authors provide exhaustive computational results of why in practice it is not easy to control the robustness to noise of the persistence signatures. Moreover, they provide additional theoretical explanations of how the stability theorem could lead to misleading claims if wrongly interpreted. Finally, the different persistent signatures are used as an input of a classifier to quantify the loss in accuracy in the noisy data.

For all these reasons, I would accept this paper after the following minor revisions.

Thank you so much for such reassuring feedback!

Reviewer #2, Comment #1

In the definition of abstract simplicial and cubical complex. The definitions are given in a very intuitive way but it would be useful to mention that they are closed under taking subsets (i.e if we have a triangle all the edges and vertices are present).

Done, in the following way:

> When the space is a point cloud $X \subset \mathbb{R}^n$, the most common choice for a structured complex is the simplicial complex, a set composed of simplices (points, line segments, triangles, tetrahedrons, and their k-dimensional counterparts, embedded in $\mathbb{R}^n$), ~~satisfying some conditions~~ that is closed under taking subsets (so that, for instance, if a triangle is in the simplicial complex, then all its edges and vertices are also elements of the simplicial complex) [8, 13].

Reviewer #2, Comment #2

It might be worth mentioning that representing each pixel with a cube is not the unique representation of an image as a cubical complex. One might also consider the dual of the complex.

Done, in the following way:

> However, a point cloud (and its simplicial complex) is not the most natural representation of an image. Indeed, this representation does not exploit the natural grid structure of images [9]. For an image Z, we can rather consider its so-called cubical complex ~~$C(Z)$~~ $K(Z)$, the cubical ~~analog of~~ analogue to a simplicial complex, in which the role of simplices is played by cubes of different dimension (points, line segments, squares, cubes, and their k-dimensional counterparts) [11]. The squares correspond to the image pixels, the edges to the sides of the pixels, and the points to the pixel corners.[1]
>
> To define a filtration function ~~$\phi : C(Z) \to \mathbb{R}$,~~ $\phi : K(Z) \to \mathbb{R}$, it is thus necessary to define the value of ϕ on each cube in ~~$C(Z)$. We avoid the details of cubical homology (to be found in [11]), and simply~~ $K(Z)$. A natural filtration function on a cubical complex assigns to each square the value of the image on the corresponding pixel, and we use $\phi(u, v)$ to denote the value of the

[1] Another way to construct a cubical complex from an image is to consider the dual of the cubical complex defined above: the points reflect the pixels, the line segments the intersections of pairs of non-diagonally neighbouring pixels, and squares reflect the intersections of four pixels [14].

filtration function on ~~a cube~~ the square corresponding to pixel (u, v). The filtration function on the line segments and points is defined as the minimum value of all bordering pixels.[2] From such a filtration function, it is straightforward to build a filtration ~~$C_{r_1} \subset C_{r_2} \subset \cdots \subset C_{r_t}$, where C_r~~ $K_{r_1} \subseteq K_{r_2} \subseteq \cdots \subseteq K_{r_t}$, where K_r is the union of all cubes corresponding to pixels (u, v) with $\phi(u, v) \leq r$ (Fig 1, top panel, and Fig 2). This family of nested subspaces is commonly referred to as the filtered cubical complex. Note that this means that (the cubes corresponding to) the pixels with the lowest filtration function value appear first and persist the longest in the filtration.

Reviewer #2, Comment #3

It might be useful adding a toy example describing a cubical filtration and what are cavities in a cubical complex.

We added the suggested figure, that we also include here for your easy reference:

Fig 2. Filtration on a cubical complex.

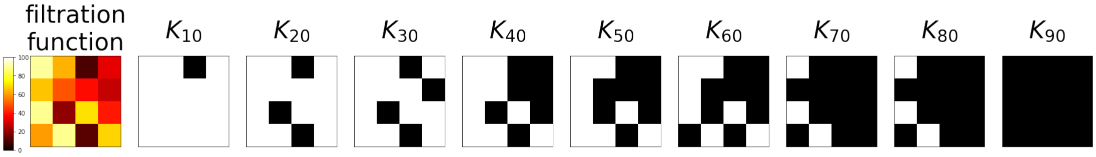

The first image represents the values $[0, 100]$ of the filtration function. The next nine figures show the cubical complexes $K_{10} \subseteq K_{20} \subseteq K_{30} \subseteq \cdots \subseteq K_{90}$, where K_r corresponds to the union of all cubes, i.e., pixels (u, v) with the filtration value $\phi(u, v) \leq r$. There is only one 1-dimensional cycle, i.e., hole (one-pixel hole in the third row and third column), which is first seen in K_{40}, and then disappears or closes in K_{70}.

Reviewer #2, Comment #4

It might be useful adding a toy example of different filtrations. For instance, an image with a heatmap representing the different types of filtrations.

We added such a figure as well, that we also include here for your easy reference:

Fig 3. Filtrations.

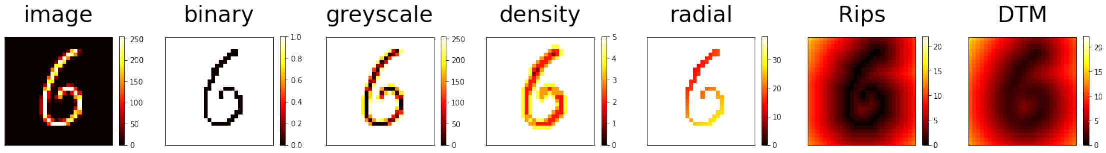

The first plot shows an example MNIST image Z, with greyscale pixel values in $[0, 250]$. The next four plots respectively show the heatmap for the binary, greyscale, density and radial filtration function $\phi : K(Z) \to \mathbb{R}$, where $K(Z)$ is the cubical complex corresponding to the given example image. The final two plots visualize the heatmap of $\phi : K(Z) \to \mathbb{R}$, where ϕ is the discretized version of the Rips and DTM filtration functions $\delta_{X(Z,z_0)} : \mathbb{R}^2 \to \mathbb{R}$ and $\delta_{X(Z,z_0),m} : \mathbb{R}^2 \to \mathbb{R}$, and $X(Z, z_0)$ is the point cloud corresponding to the image Z.

[2] A natural filtration function on the dual cubical complex assigns the pixel values as the values of the function on the points, and sets the function values for line segments and squares as the maximum value of all bordering simplices. These two methods differ with respect to the diagonally neighbouring pixels, as they are considered connected with the first approach, but not the second, what can result in substantially different persistent homology.

Reviewer #2, Comment #5

Line 161 might be misleading and line 7 in the caption of table 1. The cardinality of the persistence intervals counts the k-dimensional cycles that appear and disappear or persist along the filtration. Intervals "at infinity" count the effective numbers of cycle in the data.

The definition of the filtration function determines the "effective" cycles. Throughout our paper, the filtration functions only take finite values (note that e.g., $\delta_X : \mathbb{R}^n \to \mathbb{R}$, $\phi : K \to \mathbb{R}$, and see also Figs 3 and 4), in order to be able to calculate meaningful distances between the filtration functions (the right-hand side in the stability theorems). Therefore, the death values of any persistence intervals are also finite, so that we see no intervals "at infinity". For example, the binary filtration function ϕ_{z_0} takes only values 0 or 1, so that all cycles have a death and persistence value of 1.

We now include an additional remark:

> The cardinality of this multi-set of persistence intervals (b_i, d_i) $(i \in \mathbb{N}^+)$ counts the number of k-dimensional cycles (although many or even a majority might only show up in the filtration for a brief while, i.e., for a small range of resolution values r, yielding very short lifespans or persistence $l_i = d_i - b_i$, and as will be explained later in the paper, can thus be considered as irrelevant).

Indeed, it is only after the definition of persistence signatures and the metrics, that it is possible to properly discuss why short persisting cycles are considered less important. We had done this in the following way:

> (...) The parameter p in both W_p and l_p determines the importance of long compared to short distances.
>
> Furthermore, for a chosen p, the choice of persistence signature also influences the importance of cycle lifespans. Indeed, it is easy to see that the Wasserstein W_p^p and l_p^p distances between PDs and PLs or PIs corresponding to $PH_1 = \{(b, d)\}$ and $PH_2 = \emptyset$ reflect $(d-b)^p$, $(d-b)^{p+1}$ and $\rho^p(b, d-b)$. Since we consider $\rho(b, d-b) = (d-b)^2$ as the weight function for persistence images, this means that the cycles that persist for a short time matter the least for PIs, and the most for PDs (Table 1).

Reviewer #2, Comment #6

Line 445 449. Would add bullet points as for the other types of noise.

Done!

Reviewer #2, Comment #7

Table 5. It would be useful adding a legend to read the colors of the heatmap representing the accuracy.

Done (please see our response to your Comment #8 below)!

Reviewer #2, Comment #8

Table 5. It is not clear what are the big squares in the first row for each filtration.

We have now improved Table 5 (now Figure 7) caption to include more information:

Noise robustness and discriminative power of persistent homology on 1000 MNIST greyscale images~~, across filtrations and persistence signatures~~.

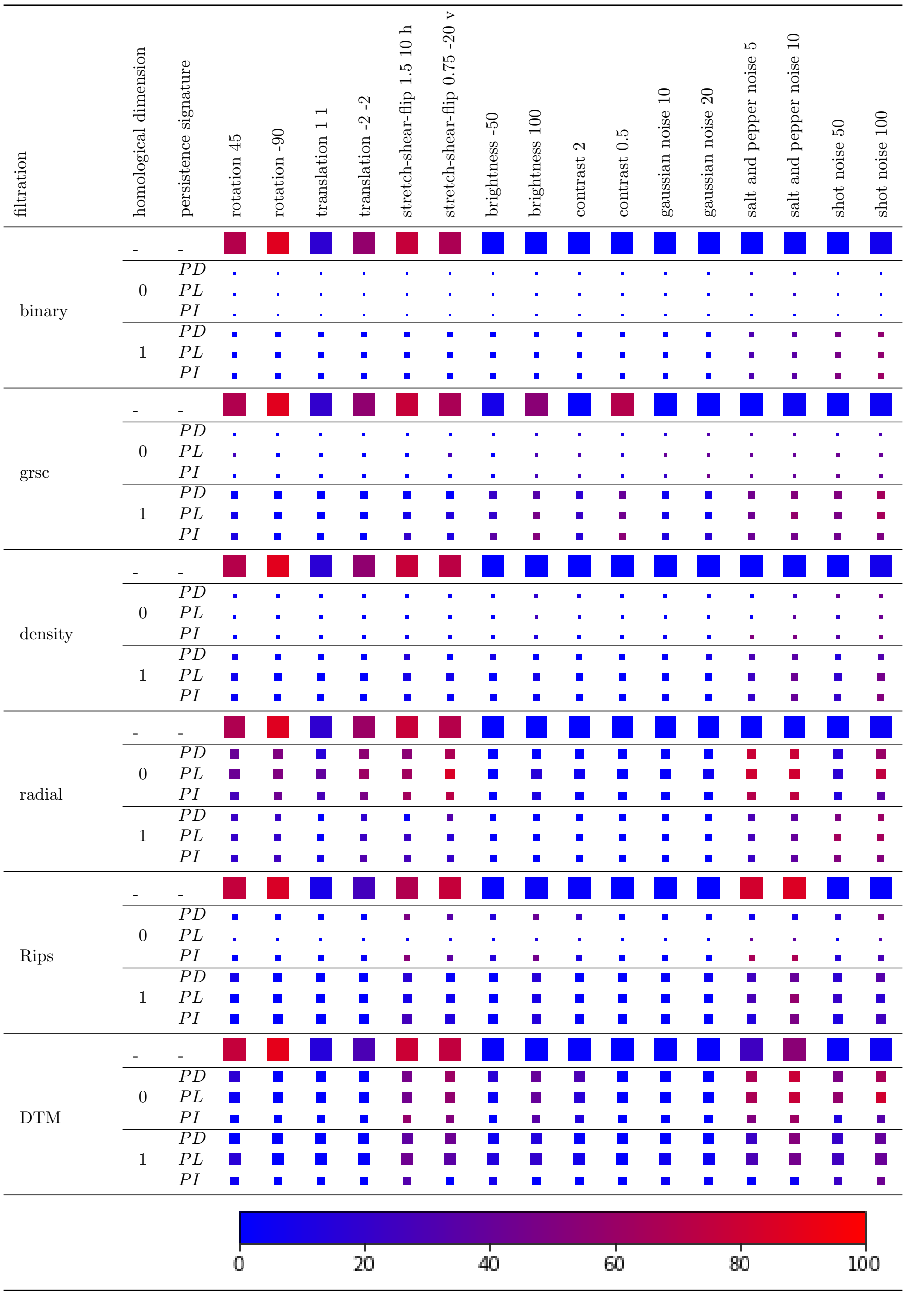

The ~~table indicates~~ figure shows the drop in SVM classification accuracy when the test dataset is noisy, compared to the non-noisy test set, averaged across 1000 images in the MNIST dataset. Each image is represented either with its filtration function values (1st row of each filtration), or with its 0- or 1-dimensional persistence diagram (2nd and 5th row), persistent landscape (3rd and 6th row) or persistent image (4th and 7th row). ~~This drop is indicated with the color of the node: blue implies 0% accuracy drop, while red indicates a significant performance loss.~~ The size of the node ~~indicates the~~ reflects the absolute accuracy on the non-noisy test data. The color of the node reflects the accuracy drop, indicated in the color bar. In particular, the presence of red nodes for PH information (2nd to 7th row) implies that PH is not robust under any type of noise, for any filtration and persistence signature.

Reviewer #2, Comment #9

Table 5 caption. It is not clear what is the meaning of the last sentence. Does it refer to the big squares in the first row for each filtration?

Please see our response to your Comment #8 above.

Reviewer #2, Comment #10

Line 564. What are the assumptions of the filtration functions?

The assumptions of the filtration functions in the stability theorems depend on a number of things, such as the values of p for the Wasserstein W_p or L_p distances, type of simplicial complex, etc. We now explicitly mention some of them:

> Firstly, stability theorems make some assumptions about the filtration function~~.~~, e.g., the function must be tame (the corresponding persistence diagram has finitely many off-diagonal points [4]), monotonic, continuous, Lipschitz or piecewise constant.

References

[1] A. Adcock, E. Carlsson, and G. Carlsson. The ring of algebraic functions on persistence bar codes. *arXiv preprint arXiv:1304.0530*, 2013.

[2] H. Anai, F. Chazal, M. Glisse, Y. Ike, H. Inakoshi, R. Tinarrage, and Y. Umeda. Dtm-based filtrations. In *Topological Data Analysis*, pages 33–66. Springer, 2020.

[3] E. Berry, Y.-C. Chen, J. Cisewski-Kehe, and B. T. Fasy. Functional summaries of persistence diagrams. *arXiv preprint arXiv:1804.01618*, 2018.

[4] F. Chazal, D. Cohen-Steiner, M. Glisse, L. J. Guibas, and S. Y. Oudot. Proximity of persistence modules and their diagrams. In *Proceedings of the twenty-fifth Annual Symposium on Computational Geometry*, pages 237–246. ACM, 2009.

[5] F. Chazal, D. Cohen-Steiner, and Q. Mérigot. Geometric inference for probability measures. *Foundations of Computational Mathematics*, 11(6):733–751, 2011.

[6] F. Chazal, B. T. Fasy, F. Lecci, A. Rinaldo, and L. Wasserman. Stochastic convergence of persistence landscapes and silhouettes. In *Proceedings of the thirtieth Annual Symposium on Computational Geometry*, page 474. ACM, 2014.

[7] Y.-C. Chen, D. Wang, A. Rinaldo, and L. Wasserman. Statistical analysis of persistence intensity functions. *arXiv preprint arXiv:1510.02502*, 2015.

[8] H. Edelsbrunner and J. Harer. *Computational topology: An introduction.* American Mathematical Society, 2010.

[9] A. Garin and G. Tauzin. A topological "reading" lesson: Classification of MNIST using TDA. In *2019 18th IEEE International Conference On Machine Learning And Applications (ICMLA)*, pages 1551–1556. IEEE, 2019.

[10] U. Islambekov, M. Yuvaraj, and Y. R. Gel. Harnessing the power of topological data analysis to detect change points in time series. *arXiv preprint arXiv:1910.12939*, 2019.

[11] T. Kaczynski, K. Mischaikow, and M. Mrozek. *Computational homology*, volume 157. Springer Science & Business Media, 2006.

[12] M. Li, M. H. Frank, V. Coneva, W. Mio, D. H. Chitwood, and C. N. Topp. The persistent homology mathematical framework provides enhanced genotype-to-phenotype associations for plant morphology. *Plant Physiology*, 177(4):1382–1395, 2018.

[13] N. Otter, M. A. Porter, U. Tillmann, P. Grindrod, and H. A. Harrington. A roadmap for the computation of persistent homology. *EPJ Data Science*, 6(1):17, 2017.

[14] P. Skraba and K. Turner. Wasserstein stability for persistence diagrams. *arXiv preprint arXiv:2006.16824*, 2020.

[15] Y. Umeda. Time series classification via topological data analysis. *Information and Media Technologies*, 12:228–239, 2017.
